# Broomrapes in Major Mediterranean Crops: From Management Strategies to Novel Approaches for Next-Generation Control

**DOI:** 10.3390/biotech14020040

**Published:** 2025-05-25

**Authors:** Demosthenis Chachalis, Eleni Tani, Aliki Kapazoglou, Maria Gerakari, Angeliki Petraki, Francisco Pérez-Alfocea, Purificación A. Martínez-Melgarejo, Markus Albert, Khalil Khamassi, Mohamed Kharrat

**Affiliations:** 1 Laboratory of Weed Science, Benaki Phytopathological Institute, 14561 Kifisia, Greece; ag.petraki@bpi.gr; 2 Laboratory of Plant Breeding and Biometry, Agricultural University of Athens, 11855 Athens, Greece; etani@aua.gr (E.T.); mgerakari@aua.gr (M.G.); 3 Hellenic Agricultural Organization-Dimitra (ELGO-DIMITRA), Department of Vitis, Institute of Olive Tree, Subtropical Crops and Viticulture (IOSV), Sofokli Venizelou 1, Lykovrysi, 14123 Athens, Greece; kapazoglou@elgo.gr; 4 Department of Plant Nutrition, CEBAS-CSIC, 30100 Murcia, Spain; alfocea@cebas.csic.es (F.P.-A.); pmelgarejo@cebas.csic.es (P.A.M.-M.); 5 Department of Biology Molecular Plant Physiology (MPP), FAU Erlangen-Nurnberg, Staudstr. 5, 91058 Erlangen, Germany; markus.albert@fau.de; 6 Field Crop Laboratory, National Institute of Agronomic Research of Tunisia (INRAT), University of Carthage, Hedi Karray St., Tunis 1004, Tunisia; khamassi.khalil@inrat.ucar.tn (K.K.); kharrat.mohamed@inrat.ucar.tn (M.K.)

**Keywords:** breeding, broomrapes, broomrape management, faba bean, hormonal interactions, tomato

## Abstract

Broomrapes (*Orobanche* and *Phelipanche* spp.) are parasitic weeds that significantly impact the productivity of major crops in the Mediterranean region, like tomato (*Solanum* spp.) and faba bean (*Vicia faba*) species. This review article extensively discusses management strategies to control broomrapes, which range from preventive measures to curative approaches. Additionally, it includes meaningful information on the intricate molecular mechanisms underlying the broomrape–host interaction, focusing on the host recognition of parasitic plant molecular patterns and the hormonal crosstalk that regulates the establishment of parasitism. Moreover, this article highlights the potential of breeding for resistance in cultivated crops, such as tomato and faba bean, as a sustainable, long-term solution to combat broomrape infestation. This review serves as a valuable resource for both researchers and farmers, offering insights for developing, implementing, and adapting effective and environmentally sustainable management practices for broomrape in Mediterranean agricultural systems.

## 1. Introduction

Broomrapes are obligate root parasitic plants (*Orobanche* and *Phelipanche* species) that can cause severe damage to many field crops. The affected crops belong to a wide range of plant families (i.e., *Fabaceae*, *Apiaceae*, *Cucurbitaceae*, *Solanaceae*, *Lamiaceae*, *Ranunculaceae*, and *Asteraceae*) [1]. Among the many broomrape species, only a limited number are considered significant threats to Mediterranean cropping systems [1,2,3,4,5]. Those include Crenate broomrape (*O. crenata*; hosts are species in *Fabaceae*, *Apiaceae*, *Cucurbitaceae*, *Solanaceae*, *Lamiaceae*, *Ranunculaceae*, and *Asteraceae*; a major problem in faba beans), small broomrape (*O. minor*; hosts are species in *Fabaceae*, *Solanaceae*, *Asteraceae*, and *Apiaceae*; a major problem in lentils, clover, and lucerne), foetid broomrape (*O. foetida*; hosts are species in *Fabaceae*; a major problem in faba beans, chickpeas, and vetch), sunflower broomrape (*O. cumana*; host is specific; a major problem in sunflower), nodding broomrape (*O. ceruna*; hosts are species in *Solanaceae*; a major problem in tomato), branched broomrape (*P. ramosa*; hosts are species in *Fabaceae*, *Apiaceae*, *Brassicaceae*, *Solanaceae*, *Cannabiaceae*, and *Asteraceae*; a major problem in tomato, tobacco, and oilseed rape), and Egyptian broomrape (*P. aegyptiaca*; hosts are species in *Brassicaceae* and *Cucurbitaceae;* a major problem in tomato, potato, lentil, and carrot). In this review, given the focus on major Mediterranean crops (i.e., cool-season legumes and industrial tomato), only four species are of great concern: *O. crenata*, *O. foetida*, *P. ramosa*, and *P. aegyptiaca*.

Broomrapes exhibit several distinctive biological and ecological characteristics that make them persistent and difficult to control. Over the last 40 years, there have been numerous publications and review papers about all aspects of the broomrape life cycle and management strategies. Some comprehensive and recent reviews are the following: [2,6,7,8,9,10]. Based on the published information, the most important characteristics of broomrapes are the following: (a) achlorophyllous nature—due to the complete absence of chlorophyll, these plants rely entirely on their host for nutrients, water, and energy; (b) specialized parasitic growth—being a root parasite, it attaches to the host roots by forming haustoria structures; (c) underground development—the majority of the broomrape’s life cycle occurs underground, and it manifests itself only in the very last stage of development, which makes it difficult to control with conventional methods; (d) prolonged dormancy—the seeds can maintain their dormancy in the soil for decades, sometimes up to 20 years, waiting for a suitable host to sprout; (e) host specificity—broomrape species tend to have high specificity for certain host plants, often parasitizing only one or a limited number of crops; (f) flowering and seed production—broomrapes are capable of producing thousands of minute seeds per plant, which can be spread by wind, water, or human activity, thus helping it spread across large areas; (g) life cycle—the seed germination is triggered by specific chemical cues from the host plant roots; (h) low success of control methods—broomrape’s underground parasitic nature makes it resilient to most traditional weed-control methods, including herbicides, mechanical weeding, and crop rotation; and (i) growth synchronization with host—there is an impressive synchronization of the parasite with the host for weed growth and development.

In the Mediterranean region, the most important winter legumes include faba beans (*Vicia faba* L.), field peas (*Pisum sativum* L.), lentils (*Lens culinaris* Medik), grass peas (*Lathyrus sativus* L.), and chickpeas (*Cicer arietinum* L.), with faba beans being the most critical. All these legumes serve as hosts to broomrape species and suffer substantial yield losses and, hence, severe economic damage due to parasitism [11]. Tomato (*Solanum lycopersicum* Mill.) is the most economically significant vegetable crop globally. Industrial tomato, in particular, is a high-value crop in the Mediterranean region, where it is severely affected by broomrape parasitism [12].

Due to the above-mentioned characteristics, broomrapes are among the most challenging agricultural problems to tackle that could threaten major Mediterranean crops. Despite extensive efforts over the past five decades, broomrape management has often been ineffective, leading to continuous spreading into new growing areas [10]. Novel approaches to understanding the mechanism of host–parasite interaction and concentrated efforts to develop resistant cultivars are highly needed more than ever before.

In this review, all the latest research knowledge is presented in the following sections: preventive and curative strategies for broomrape management, hormonal interactions related to host signal perception and haustorium formation, molecular mechanisms through which parasitic invaders are recognized by host plants, and breeding efforts aimed at the development of resistant cultivars. The focus is given to *Orobanche* and *Phelipanche* species that parasitize major Mediterranean crops (i.e., cool-season legumes, with an emphasis on faba beans and industrial tomato), although the information presented here could be beneficial regarding other host crops, because either similar control methods exist or host–plant interactions may be similar.

## 2. Management Strategies

Significant research efforts to manage broomrape infestations in major Mediterranean crops have been undertaken, with approaches ranging from agronomic practices to biological control methods. Despite these efforts, the efficacy levels remain largely unsatisfactory, and the problem persists in agricultural practice (Table 1). Although significant scientific discoveries have been made in the process, these measures have often had limited success in terms of commercial application. The limited success of these approaches can be mainly attributed to (a) partial protection, because most solutions have only provided partial protection against broomrape; and (b) low economic viability, because many of these solutions are not economically feasible, particularly for low-input legume cropping systems. Managing broomrape (*Orobanche* spp.) effectively requires a combination of preventive and curative measures that aim to reduce the risk of infestation and minimize its impact on crops.

### 2.1. Preventive Measures

Broomrapes are parasites that rely almost totally on their hosts for their survival, and, hence, integrated management strategies focusing on preventing their establishment and spread are essential. Below are presented the key preventive measures for the effective management of broomrapes.

#### 2.1.1. Crops’ Exploitation

Crop rotation, which reduces the frequency of host cultivation, is one of the primary strategies farmers use to manage broomrape infestations. The key strategy is to rotate susceptible with non-susceptible crops, aiming to reduce the weed’s impact over time. This key strategy is served by the following three pillars: (a) lower the presence of host crops, which could limit broomrapes, reducing their opportunities to parasitize and spread; (b) prolong soil seed dormancy, because the broomrape cannot parasitize or germinate in the absence of a suitable host; and (c) limit soil seed bank replenishment, as no seed rain can come from newly developed broomrape plants. Rotation, however, is a long-term strategy due to the long viability of the seed bank [13], and it requires at least a five-course and, ideally, a nine-course rotation [14,15,16,17]. In such a rotation scheme, non-host crops and fallows should be included to prevent broomrape seed bank increases and manage broomrape outbreaks. In this context, cereals (corn, wheat, and barley) or grass could be included to manifest a direct and indirect impact on parasitism. Previous research has shown that good rotation crop examples were alfalfa (*Medicago sativa*), wheat (*Triticum* spp.), and cultivated oat (*Avena sativa*) [18,19]. In addition, where severe broomrape problems exist in cool-season legumes (i.e., in Ethiopia), other rotational crops such as common beans (*Phaseolus vulgaris* L.) and fenugreek (*Trigonella foenum-graecum* L.) crops are selected [9]. The impact of rotation could be enhanced by incorporating trap, catch, or allelopathic crops into the scheme, as suggested by studies like Habimana et al. in 2014 [20].

Trap crops are plant species whose root exudates strongly stimulate broomrape seed germination, yet they do not become infected, as they are resistant to the subsequent stages of parasitism. This ultimately results in the death of young broomrape seedlings due to the absence of a suitable host. Several promising trap crop examples have been identified by screening root exudates from various important crop species [13]. Indicatively, trap crops for *P. ramosa* include flax, phaseolus bean, sorghum, maize, sunflower, and cucumber [21,22]. Additional trap crop examples include coriander (*Coriandrum sativum* L.), basil (*Ocymum basilicum* L.), fenugreek (*T. foenum-graecum* L.), and clover (*Trifolium alexandrinum* L.) [4,20,23]. Recent reports have shown that intercropping flax or fenugreek with faba bean cultivars significantly decreased the levels of *O. crenata* infection, while it increased the faba bean yield compared with sole treatments; flax more effectively reduced the infection rate of *O. crenata* compared to fenugreek [24]. However, there are only a very few good case studies of trap crops; therefore, this strategy should be considered only as a component of an IPM approach.

Catch crops could induce high broomrape germination, but the progression of parasitism does not materialize due to mechanical, physiological, and chemical factors. Catch crops are mostly short-term crops that are harvested or destroyed shortly after sowing (i.e., after 6–8 weeks), mainly at the vegetative stage and prior to parasite emergence or before parasite flowering [25]. In legumes, tori (*Brassica campestris* var. *toria*), when effectively managed as a catch crop, can lead to a reduction of up to 30% in the broomrape seed bank [26]. According to Nadal et al. (2005) [27], using faba beans as a trap crop (i.e., harvested before broomrape can flower and produce seeds) can significantly deplete the broomrape seed bank by more than 30% in a single season. In industrial tomato, flax could be an example of a catch crop; the crop was sown 4–6 weeks prior to tomato transplantation, and it significantly suppressed *P. ramosa* infestation [6]. Other good examples are *Vigna unguiculata*, *Hibiscus sabdariffa*, *Hordeum vulgare*, *Sorghum bicolor* [7], and *Lotus corniculatus* [28]. A large screening survey of several plant species, including summer and winter plants, medicinal herbs, and important weed species, for trapping and catching species for *P. ramosa* was conducted [29].

Allelopathic crops could play a role in managing broomrape populations by preventing seed germination, disrupting attachment, and inhibiting growth. These crops could produce allelochemicals that are secondary metabolites that would manifest a variety of effects, both on the parasitic plants themselves and on the surrounding ecosystem. Allelopathic crops have low efficacy on their own and, ideally, should be part of the rotational scheme or other IPM practices. A good example of such an allelopathic crop in legumes is fenugreek (*Trigonella foenum-graecum* L.) [30]; trigoxazonane was identified from fenugreek root exudates, which would be responsible for the inhibition of *O. crenata* seed germination [31]. Another allelopathic crop could be *Desmodium uncinatum*, which was shown to inhibit the development of *Striga haustoria* [32]. A number of compounds like vanillic, o-coumaric acids, or scopoletin that are excreted from cereals’ roots [33] have allelopathic effects [34]; thus, cereals could serve as key allelopathic crops in managing broomrapes. The complex nature of both the direct and indirect effects of allelochemicals on parasitic plants poses significant challenges for their effective use in control strategies.

Winter cover crops could serve as an important alternative solution for suppressing parasitism outbreaks in summer crops such as industrial tomato; in cool-season legumes, they have no practical value. Little research has been conducted to document the effects of winter cover crops in managing broomrapes. These service crops provide several benefits, including weed control, improving soil health, and reducing broomrape seed bank buildup. Winter cover crops are planted in the off-season, typically between harvest and the next planting season, and can suppress broomrape growth through several mechanisms as catch, trap, or allelopathic crops, as previously discussed. In this context, Brassica species release glucosinolates [35]; common cereals’ (i.e., rye) root exudates could have an allelopathic effect on broomrapes [36]. Haidar et al. (1995a) [37] reported that surface-applied and pre-plant incorporated wheat and barley straw mulch residues presented to have a significant impact on the infestation reduction and growth of *P. ramosa* in potatoes. Utilizing cover crops by adding dried powdered leaves of radish (*Raphanus sativus*) to the soil can be an effective method for reducing the incidence of *P. ramosa* and protecting tomato plants (*S. lycopersicum*) from parasitism [38]. Using cover crops to manage broomrapes could be part of the IPM strategy [39], but it requires careful selection of the cover crops and consideration of the relationship between these plants and the parasitic broomrapes.

#### 2.1.2. Modifications to Sowing and Transplanting Dates

The modification of sowing in legumes and transplanting in industrial tomato could help to manage broomrape outbreaks. It is known that key environmental factors (i.e., soil temperature and moisture) are critical for the onset of parasitism [40]. In this context, the modification of dates could affect all three important stages: preconditioning, germination, and attachment/development of parasitism.

In faba beans, previous reports have shown that one-month shifting (from October to November and from December to January) reduced *O. crenata* and *O. foetida* parasitism [14]; similar results were demonstrated by Perez-de-Luque et al. in 2004 [41]. Also, in other field trials in Ethiopia, delayed-sown faba beans had lower levels of parasitism [42]; similar results have been demonstrated in chickpeas [43]. Recent reports have shown that a 3-week delay in faba bean sowing can lead to a significant reduction in the number of emerged *O. crenata* shoots for both resistant (Giza 843) and susceptible (Nubaria 1) cultivars [24].

In industrial tomato, previous reports have shown that earlier transplanting periods manifested higher yields and a lower concentration of broomrape [44]. However, the modification of dates could have a serious negative effect on crop development and final yields; delayed legume sowing might create higher abiotic stresses (i.e., heat stress in flowering or drought situations), whereas early transplanting in industrial tomato might subject the crop to suboptimal growing conditions. In addition, the modification of dates could benefit scenarios with low or moderate levels of parasite infection [45].

#### 2.1.3. Solarization

Soil solarization has been shown to be an effective non-chemical method in reducing broomrape seed viability in areas with sufficiently hot summer climatic conditions. Therefore, this method is more suitable for cool-season legumes but lacks practical value for industrial tomato, as it would necessitate leaving tomato fields fallow for several months during the Mediterranean summer growing season. In general, solarization can radically raise top soil temperatures up to 15 cm; soil should be moist and covered with transparent polyethylene sheets for at least 1–2 months. Older reports have demonstrated that soil solarization using polyethylene sheets to cover moist soil for several weeks under sunlight can effectively control *Orobanche* seeds present in the upper soil layers [46]. Several other reports confirmed the significant effects of soil solarization in killing *P. ramosa* seeds [21,47,48]. Additionally, the great benefits of soil solarization (99% death of viable broomrape seeds in the seed bank) were documented when it was applied for two consecutive summers without any negative impact on tomato yield [47]. The major drawbacks of soil solarization are the following: the high cost of implementing the method, the need to preserve soil moisture during the hot summer period, and the lack of irrigation for cool-season legumes. All the above restrictive elements undermine the feasibility of applying this method despite its high efficacy in controlling broomrape parasitism.

#### 2.1.4. Tillage, Mechanical Cultivation

Tillage and mechanical cultivation could affect the weed soil seed bank, including broomrape seeds. Conventional tillage (i.e., moldboard plow), although it may reduce germination stimulation, finally results in the enhancement of the existing soil seed bank, as broomrape seeds can potentially be viable for more than a decade [49]. In addition, they contribute to a higher seed persistence level by protecting seeds from aging, predation, and induced dormancy caused by lower oxygen levels found in deeper soil layers [50].

Two distinct non-conventional soil management practices could have an impact on controlling broomrape infestation: minimum tillage and deep ploughing. Previous reports showed that the lowest tillage decreased the amount of viable broomrape seeds incorporated in soil [51] and, hence, their potential germination. On the other hand, deep ploughing is a technique often recommended to control broomrape that involves burying seeds at a depth where they cannot germinate as a result of insufficient oxygen or unfavorable conditions [52,53,54]. Finally, both minimum tillage and deep tillage, although they could have moderate efficacy, seem to be of lower agronomic value in practice given their feasibility limitations.

#### 2.1.5. Fertilization

Proper nutrient management can help make the soil less conducive to the establishment of parasitism in crops. Fertilization can influence the soil and plant environment in ways that reduce the likelihood of broomrape seeds attaching to host plants and initiating parasitism in clover and tomato [55]. Nutrient management plays a crucial role in promoting tolerance against broomrape parasitism in different crop developmental stages and in the pre- or post-attachment stages of the parasite [56].

Direct exposure to fertilizers like urea and ammonium can be toxic to broomrape, suppressing both seed germination and seedling development [11]. Also, the negative effect of ammonium on broomrapes is linked to the plant’s limited ability to detoxify ammonium using the enzyme glutamine synthetase [2]. However, other forms (i.e., nitrates) did not exhibit the same inhibitory effects [55,57]. In addition, fertilization could modify the synthesis and exudation of root stimulants (i.e., strigolactones) of the host crop. Phosphorus and nitrogen have been reported to downregulate strigolactone exudation in several crop host species [58]. In industrial tomato, given the fertigation system, it could be important to utilize the fertilization schedule in managing broomrape invasions, whereas in legumes, this would be considered of lesser importance.

#### 2.1.6. Use of Synthetic Stimulants, Suicidal Germination

This method involves the promotion of suicidal germination in broomrape (*Orobanche* spp.) through the use of synthetic molecules that mimic the natural germination-inducing factors released by host plants, such as strigolactones [59]. This technique is based on the idea of tricking the parasite into germinating in the absence of a suitable host, thus leading to the death of the parasite seedling because it is unable to acquire the necessary nutrients through parasitism. Previous research on the direct application of synthetic strigolactone analogs (i.e., GR7) to deplete the soil seed bank [59] proved very challenging due to instability issues and the difficulty of achieving consistent and effective application in the field [60]. Newer types of synthetic strigolactone analogs (i.e., Nijmegen-1) that were more stable and better suited for field applications resulted in high suicidal potential [61]. Research has shown the complex interactions between strigolactones and DNA methylation and the potential of using synthetic compounds to control *P. ramosa* [62]. In addition, recent studies have demonstrated the potential of auxin-derived strigolactone mimics to induce suicidal germination in broomrapes [63]. Other types of stimulants include triazolide strigolactone mimics [64] and phthalimide-derived strigolactone mimics [65], which have been synthesized as potent selective germinators of broomrape seeds. However, for this method, significant challenges exist, such as variable efficacy levels, low stability, high production costs, and potential off-target effects in the soil microorganisms.

#### 2.1.7. Polyethylene Plastic Mulching

Polyethylene sheet mulching is a cropping system that is used in industrial tomato because it offers significant benefits such as early planting, yield increases, better agrochemical utilization, and enhanced weed control. Regarding broomrape control, the use of polyethylene sheets acts by modifying the temperature and moisture in soil. The plastic sheet can significantly increase the soil temperature underneath it, creating an unfavorable environment for broomrape seed germination and root attachment. Enhancing and preserving high soil moisture could make it more challenging for broomrape seeds to establish a successful parasitic relationship with the tomato plants. Research demonstrated that black polyethylene sheeting applied on the day of transplanting tomato completely controlled broomrape in the field by preventing seeds from germinating and attaching to the plant roots [66]. Similarly, polyethylene mulch application had the highest efficacy in controlling broomrape in aubergines, with greater than 95% control. The polyethylene mulch likely prevented broomrape seed germination and created an unfavorable environment for seed attachment and development [67]. This method has significant constraints and limitations, such as a smaller scale of potential growing areas; the high cost of plastic sheets, equipment, and labor; and the environmental impact of plastic disposal or recycling.

#### 2.1.8. Use of Crop Tolerance Inducers

Host plants could employ systemic acquired resistance (SAR), that is, a plant’s defense mechanism that is triggered by an initial localized infection or injury, leading to the activation of defense responses throughout the entire plant to defend against parasitism. As such, salicylic acid and benzothiadiazole application has activated broomrape resistance in various legumes such as faba bean [68], pea [41], and red clover [69].

In tomato, both salicylic acid and indole acetic acid can activate the defense mechanisms against *P. ramosa* parasitism [70]. Priming tomato seeds with different concentrations of the above hormonal inducers significantly reduces the number and biomass of *Orobanche* spp. tubercles and improves tomato root growth [71]. Based on this principle, experiments have shown that a commercial product (i.e., Bion^®^), under experimental conditions, could decrease broomrape infection by 80% in hemp and tobacco [72]. Other inducers were also investigated, such as L-methionine, that have the potential to reduce broomrape invasions on tomatoes [73]. Research has demonstrated the role of biotic inducers of systemic resistance (ISR) in enhancing a plant’s defense against broomrape parasitism. Specifically, *Rhizobium leguminosarum*, a type of beneficial bacterium, has been shown to induce defense mechanisms that help protect plants from broomrape infection [74]. Additionally, strains of Pseudomonas sp. were documented to trigger induced systemic resistance [75], and a commercial product (i.e., Proradix^®^), in experimental conditions, showed high (80%) control of broomrape parasitism [72]. However, no field applications of the above methods have been tested under real field conditions.

#### 2.1.9. Use of Resistant/Tolerant cvs

While significant research efforts have been made to develop resistant or tolerant cultivars to broomrape in both legumes and industrial tomatoes, success has been more pronounced in legumes, particularly in faba beans, where a few moderately successful examples of broomrape resistance/tolerance have been achieved. By contrast, there has been no commercially successful broomrape-resistant cultivar developed for industrial tomatoes. ICARDA has developed a multi-site screening program aiming to develop cultivars with resistance to broomrape [76] using as the main source of Orobanche resistance the line F402, which was identified in the early 1970s [77], and some minor sources available in different Mediterranean countries. In this context, over the last 40 years, a number of faba bean cultivars with resistance/tolerance to broomrapes have been developed, such as Baraca, Quijote, Faraon, Navio, Quijote, Najeh, Chourouk, Misr1, and Giza843 [78,79,80,81]. Some of these cultivars (i.e., Quijote, Navio, and Najeh) are notable for their unique and effective resistance mechanism against broomrape through a mechanism that involves reduced strigolactone exudation from their roots [82].

Finally, substantial research is needed in this direction to identify a range of broomrape-resistant cultivars with different precocities that give farmers the flexibility to select the best-performing varieties for their specific environmental and farming conditions. This approach maximizes the potential for controlling broomrape and enhances the sustainability and productivity of agricultural systems by allowing for adaptation to diverse growing conditions and crop management strategies. A full account of the latest developments in breeding for resistance in both faba beans and industrial tomatoes is presented in the following sections of this review.

### 2.2. Curative Measures

Curative measures to manage broomrapes are generally applied after the parasite has already established itself in the field. Unlike preventive measures, which aim to prevent infestation, curative measures focus on reducing the impact of existing broomrape infestations or attempting to eliminate or control the parasite after it has been established in crops.

#### 2.2.1. Seed Dressing Treatments

Previous research in faba beans has shown that seed dressing treatments with imidazolinones proved effective for controlling *O. crenata*; additionally, moderate control using imazethapyr was measured in faba bean and pea seed treatments [83]. In tomato, seed dressing using sulfonylureas (i.e., chlorsulfuron and triasulfuron) in low doses showed some effect on broomrapes, with good safety for the crop [84]. However, seed dressing treatments have a low residual effect and need to be supported by other herbicide applications to show their full effectiveness [4].

#### 2.2.2. Foliar Herbicides

In cool-season legumes, being a low-input rain-fed cropping system, only systemic herbicides can be used by foliage spraying, absorption, and translocation of the herbicide to the roots where broomrape germination and establishment are occurring. In this context, the use of glyphosate with lower doses (at 60–120 g a.i./ha) has been shown to effectively control broomrapes in faba beans, lentils, and peas [9,23,85]. In addition, imazethapyr, imazapyr, and chlorsulfuron were effective in controlling broomrapes (i.e., *O. crenata*, *P. aegyptiaca*, and *O. foetida*) in legumes [86,87].

In industrial tomato, although lower doses of glyphosate (i.e., 30 to 50 g a.i./ha) were effective against *P. ramosa* and *P. aegyptiaca*, they significantly affect crop development and reduce tomato yields [7,88]. In addition, several sulfonylureas (i.e., bensulfuron, chlorsulfuron, nicosulfuron, primsulfuron, trimsulfuron, thifensulfuron, and triasulfuron) have been shown to have a variable level of effectiveness in broomrapes (i.e., *O. aegyptiaca*) under real field conditions [89]. Also, imazethapyr applications, although very effective in broomrape control, caused severe phytotoxicity problems in tomatoes [70].

#### 2.2.3. Herbigation

Herbigation (i.e., the delivery of herbicides through irrigation water) is a method that could be employed in industrial tomato, targeting broomrape seeds and young attachments. Previous research has shown that herbigation with some sulfonylurea herbicides (i.e., sulfosulfuron, chlorsulfuron, triasulfuron, rimsulfuron, imazapic, and imazamox) could be used to control broomrapes (i.e., *P. aegyptiaca* and *P. ramosa*) in tomato through the soil [7,90]. In this context, the most effective herbicide was found to be sulfosulfuron, applied at rates of 37.5–75 g a.i./ha, to control broomrapes (*P. aegyptiaca*) at a maximum soil depth of 18 cm [50]. However, herbigation effects showed a range of efficacy levels (from low to high) due to several complex factors such as the herbicide’s residual effect, the precise identification of the right developmental stage of the parasite, the type of irrigation water application, repeated applications, and accurate dose application with irrigation [89].

#### 2.2.4. Biological Control

Biological control presents a promising and environmentally sustainable strategy for managing broomrapes in field crops; the high specificity of such agents is one of the key advantages, as this selectivity allows the targeted suppression of the parasite without harming the crop plants [91]. There has been a plethora of published information showing that several *Fusarium* spp. and other plant pathogens have the potential to attack various species of broomrapes in field crops [7].

*Fusarium oxysporum* and *Neocosmospora solani* (*synonyms Nectria haematococca*, *Haematonectria haematococca*) are among the *Fusarium* species that are the most promising because of their rapid growth, intensive sporulation, and chlamydospore formation [92]. Other *Fusarium* species include *F. venenatum* isolates for tobacco [93] and *F. acuminatum*, *F. fujikuroi*, *F. proliferatum*, and *F. solani* for various crops [94]. Other reports have indicated that *Alternaria alternata*, *Dendrophora* sp., *Chaetomium* sp., and *Talaromyces trachyspermus* could be potential good agents for controlling broomrapes [7]. Despite the huge research effort that has been undertaken in the last 40 years, the development of effective biological controls for broomrape remains an ongoing challenge, with the poor field efficacy of known fungal pathogens being the primary barrier to commercialization. However, through approaches such as improving pathogen virulence, optimizing inoculation techniques, and incorporating biocontrol into IPM systems, it would be possible to overcome these challenges [95].

#### 2.2.5. Beneficial Insects

From the long list of 40 species from 22 families of beneficial insects that could be used as biocontrol agents [96], only a limited number proved to be good case studies, such as *Phytomyza orobanchia* Kalt., *Chyliza extenuata*, *Diaphora mendica*, and *Celypha* spp. [97]. The most promising beneficial insect for controlling broomrapes is *Phytomyza orobanchia*; old reports have shown its ability to control *O. cumana* (sunflower broomrape) and *O. cernua* [98]. In the last decades, significant research has shown its potential to control broomrapes in cool-season legumes, mostly in faba beans, by parasitizing seed capsules, which causes a great reduction in broomrape seed production ranging between 29 and 94% depending on the *Orobanche* species [70]. While *Phytomyza orobanchia* holds promise as a biocontrol agent for *Orobanche* species, the reality is that its widespread natural distribution and its limited impact on seed production in high-infestation areas make it an imperfect solution for broomrape control. Despite extensive study and attempts at breeding and release, the insect has not demonstrated sufficient efficacy to warrant a commercially viable product.

#### 2.2.6. Hand Weeding

Hand weeding is often the first and most straightforward measure mentioned for controlling broomrape species in field crops, especially when infestations are still light. This manual method involves the physical removal of emerged broomrape plants from the field, followed by their destruction. The impact of hand weeding is minimal on broomrape competition to the host crop but aims to reduce the soil seed bank. In industrial tomato, its feasibility is low, given the dense canopy that covers the soil and makes surveillance and removal very laborious. As such, hand weeding can be effective in small-scale or early-stage infestations; it comes with several limitations and challenges that make it not feasible for large-scale farming.

## 3. Recognition of Parasitic Plant Molecular Patterns by Hosts

To protect themselves from pathogenic invaders, plants possess passive defenses, such as the formation of physical barriers (cuticles, lignified cell walls, etc.) or the storage and release of secondary compounds. In addition, plants have evolved an innate immune system to detect pathogens and actively fend them off. The main precondition of plant immunity is pattern recognition receptors (PRRs) [99,100,101,102] that recognize herbivore-, microbe-, or generally pathogen-associated molecular patterns (HAMPs, MAMPs, and PAMPs, respectively) [103,104]. Well-known PAMPs include molecules such as chitin of fungal and arthropod origin, flagellin and other proteins of microbial origin, or other compounds like lipopolysaccharides and cell wall fragments. PAMPs are detected by plant pattern recognition receptors (PRRs) at extremely low concentrations (nano- to picomolar levels), initiating a range of defense responses such as calcium influxes, ethylene production, the synthesis of secondary metabolites (e.g., callose, phytoalexins, and lignins), the accumulation of reactive oxygen species (ROS), and the activation of defense-related gene expression [104]. Collectively, these cellular defense responses limit pathogen development and can lead to the partial or even complete resistance of the host plant to the invading pathogen.

Unlike microbial plant pathogens, parasitic plants are angiosperms and often closely related to their hosts, which makes it more complex for host plants to recognize them as alien invaders. Distinct molecular patterns that differentiate parasitic plants from the “self” are likely to be rare and must exhibit high specificity. To date, only a few plasma-membrane-bound PRRs have been identified in host plants that recognize parasitic plants and induce defense mechanisms that help to restrict parasitic plant growth. For example, one gene in sunflower that presents resistance to *O. cumana* races was identified using a map-based cloning strategy. This gene, *HaOr7* (*Helianthus annuus Orobanche* resistance 7), encodes a leucine-rich repeat receptor kinase (LRR-RK) that locates to the plasma membrane, and the complete HaOr7 protein appears to be present exclusively in *O. cumana*-resistant cultivars of sunflower [105]. Susceptible cultivars include a variant of the gene that encodes a truncated HaOr7 protein, lacking the kinase domain of the LRR-RK HaOR7, and, therefore, seems to be not functional. In cases of successful resistance, *O. cumana* may initially penetrate sunflower root tissues; however, the formation of a vascular connection within the central cylinder is prevented by resistance responses triggered by the *HaOr7* gene [105].

Recently, a leucine-rich repeat receptor-like protein kinase has been identified that enhances tomato resistance to *P. aegyptiaca* [106]. In that study, field, pot, and rhizotron experiments were conducted to assess 118 tomato varieties against *P. aegyptiaca*. Further proteomic research, genome-wide analysis, and genetic engineering were performed to determine the *LRR-RLK* genes that control tomato resistance to *P. aegyptiaca*. One resistant variety (‘H1015’) was identified, which manifested slower growth, partial necrosis, and a lower number of successfully infecting parasites compared to the susceptible variety (‘H2401’). The authors finally identified LRR-RLK-3 as the essential key player involved in resistance mechanisms. However, the protein LRR-RLK-3 is not a typical LRR-RLK, as it lacks the kinase domain as well as a transmembrane helix domain. Subcellular localization of this truncated protein, which appears to be a soluble LRR domain, seems also to not be in the tomato plasma membrane but rather in the ER/nucleus [106]. A binding partner or PAMP of parasitic *P. aegyptiaca*, as well as a mechanistic mode of action for SlLRR-RLK-3, still must be elucidated.

Parasites of the genera *Cuscuta* comprise about 200 species that all infect the above-ground parts of hosts, preferentially the stems. However, the cellular resistance mechanisms in the host plants show similarities in terms of the mechanisms and signaling pathways to those triggered by the root-parasitic *Orobanchaceae*. Remarkably, some species of the Solanaceae show an active defense reaction against selected Cuscuta species [107]. Indeed, the observation of the resistance of different tomato species against diverse Cuscuta species unraveled a phenomenon comparable to “race-specific resistance”, following a concept described for gene-for-gene interactions. For example, the strong resistance reaction of cultivated tomato (*S. lycopersicum*) seems very specific to *Cuscuta reflexa* [108], and several other Cuscuta species can successfully infect tomato, including *C. pentagona*, *C. campestris*, *C. suaveolens*, and *C. europaea* [109,110].

The resistance response of cultivated tomato species against *C. reflexa* is visible right at the haustorium penetration sites [108,111]. About three to five days after the prehaustorium development, in the late attachment phase, epidermal host cells at the contact sites elongate and burst [112,113]. Sub-epidermal cell layers secrete soluble phenylpropanoids and show a boosted activity of peroxidases. Along with synthesized di-fatty acids, omega-di-OH-fatty acids, and lignin, these enzymatic activities facilitate the crosslinking of cell wall compounds and enhance the formation of a suberin-like barrier that blocks the haustoria penetration of *C. reflexa* in tomato roots [107].

**Figure 1:**
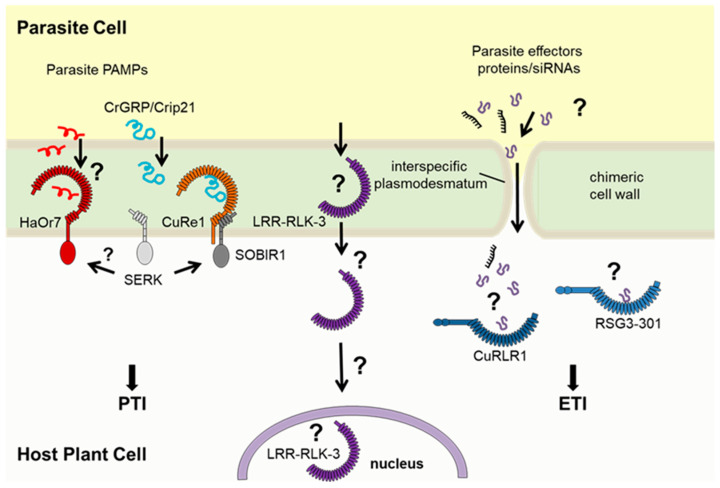
PAMPs and recognition by PRRs in plant–parasitic plant interaction. Pathogen-associated molecular patterns (PAMPs) of parasitic plants, like *C. reflexa* GRP or its peptide epitope Crip21 [119], or other yet-unknown patterns are documented at the host cell surface by membrane-bound pattern recognition receptors (PRRs), such as HaOr7 [105], CuRe1 [114], or tomato LRR-RLK-3 [106], and induce PAMP-triggered immunity (PTI) in host plant cells. Some host plants possess resistance proteins such as CC-NBS-LRR proteins that are located to the cytosol and may recognize effectors intracellularly. Consequently, those types of receptors induce effector-triggered immunity (ETI), often accompanied by cell wall lignification or a hypersensitive response, leading to resistance against parasitic plants; modified from Albert et al. 2021 [123].

The way that these active tomato resistance responses are switched on is only partially understood. One resistance-related gene was mapped on tomato chromosome 8 in an introgression line (IL)-based mapping approach [114]. Because *S. lycopersicum* shows an active defense response against *C. reflexa*, whereas the tomato relative *S. pennellii* is fully susceptible, the genetic diversity has been explored, and a collection of *S. lycopersicum x S. pennellii* ILs [115,116] has been used to map the tomato defense against *C. reflexa*. The identified tomato gene encodes an LRR receptor protein (LRR-RP)—Cuscuta receptor 1 (CuRe1)—which plays a critical role in the recognition of *C. reflexa* and in the stimulation of defense-related responses [114]. CuRe1 is a plasma membrane-bound receptor that lacks an intracellular kinase domain. To switch on cellular signaling, it constitutively exists in a heteromeric complex with the adaptor kinase SOBIR1, a requirement that has been described for LRR-RLPs in general [117,118]. CuRe1 is a receptor in tomato plants that recognizes a 116-amino-acid glycine-rich protein (CrGRP) from the cell wall of the parasitic plant *C. reflexa* as a PAMP. A smaller 21-amino-acid peptide fragment called Crip21 specifically binds to CuRe1 and triggers defense responses [119]. Both CrGRP and Crip21 induce classic PAMP-triggered immunity (PTI) responses, such as reactive oxygen species (ROS) bursts and ethylene production [114,119,120]. When CuRe1 is introduced into normally susceptible host plants, it contributes to resistance and reduces C. reflexa growth. However, CuRe1 is not the only gene involved in tomato resistance; other factors, likely additional receptors, also contribute to full resistance. For example, loci mapped to chromosomes 1, 2, and 6 in an IL-based screen also appear to be critical for tomato resistance against *C. reflexa* [121]. Although CuRe1 is a plasma membrane-bound receptor and belongs to the classical set of PTI-related components, its contribution to mechanisms such as effector-triggered immunity (ETI) is possible. Indeed, a CC-NBS-LRR (coiled-coil-nucleotide-binding site-LRR) protein has been acknowledged that appears to play a fundamental role in the lignin-based resistance of *Solanum habrochaites* against *C. campestris* [122]. This cytosolic receptor, termed CuRLR1 (Cuscuta Receptor for Lignin-based Resistance 1), likely recognizes unknown parasitic effectors and works together with transcription factors to confer host resistance. In resistant plants, the stem cortex undergoes localized lignification upon *C. campestris* attachment, blocking parasite penetration (Figure 1).

For Striga–host interactions, a race-specific resistance has also been described, following in principle the mechanism observed for ETI [123]. *S. gesnerioides* races have been categorized according to their genetic relatedness and capacity to differentially parasitize cowpea varieties and landraces [124], and various race-specific resistance genes have been mapped in the cowpea genome [125]. Using a molecular marker-assisted positional cloning strategy, ref. [126] subsequently isolated the *RSG3-301* gene from cowpea that confers resistance to *S. gesnerioides* race SG3 and showed that it encodes a typical CC-NBS-LRR protein. The characterization of RSG3-301 led to the suggestion that race-specific Striga resistance in cowpea is an example of effector-triggered immunity (ETI) in which intracellular NLR proteins (such as RSG3-301) are stimulated either directly or indirectly upon recognition of pathogen/parasite effectors [127].

## 4. Hormonal Crosstalk Between Crop and Parasite

As has already been mentioned, *Phelipanche* and *Orobanche* spp. develop the haustorium after recognizing hormonal signals released by the host [128]. Various studies demonstrate that such holoparasites have adopted the ability to detect the same signaling molecules as other beneficial organisms, such as arbuscular mycorrhizal fungi (AM), and trigger the germination of their own seeds in the vicinity of host roots. Direct physical connections between the two ensure the unidirectional transfer of resources (water and nutrients) from host to parasite but also the exchange of small signaling molecules, such as plant hormones, that concomitantly shape the metabolic activity of both partners [129,130]. Advances in understanding this signaling interaction between host and parasitic plants have significantly enhanced our knowledge of the evolution of plant parasitism. These insights are also aiding in the development of more effective control strategies against parasitic plants that have become major threats to important agricultural crops such as sunflower, tomato, tobacco, potato, and rapeseed [131]. However, despite advances in understanding these interactions, a significant challenge remains in determining whether parasitic plants exude hormonally active metabolites in the rhizosphere, although there are indications that this may be the case [129,132,133]. In the present review, we provide a bibliographic overview, including the latest findings on hormonal interactions related to host signal perception and haustorium development, where much remains to be deciphered.

Strigolactones (SLs) are the most well-studied class of germination stimulants for holoparasitic seeds. While angiosperms require optimal environmental conditions of light, temperature, and moisture for germination after dormancy release, parasitic plant seeds require the presence of SLs as a chemical signal indicative of host proximity. Additionally, the concentrations of SLs required to induce germination in parasitic plants differ from pM to μM, depending on the strigolactone, likely due to differences in the germination-stimulating receptor or subsequent signaling, possibly reflecting the coevolution of the parasitic plant species with their hosts [134]. The synthesis and exudation of these key chemical signals in parasitism are finely modulated by other biotic factors and changing environmental conditions [129]. The reduction in SL release by infested host roots could be an adaptive strategy to suppress parasitic seed germination [135,136]. However, because SLs are also a plant hormone that regulates many aspects of plant development, SL deficiency may also impact the post-germination stages of the parasite–host interaction. In this context, SL-deficient tomato mutant plants (SlCCD8 RNAi) infected with pre-germinated seeds of Phelipanche exhibited higher levels of infection and faster parasite development, which suggests that SLs play a positive role in host defense against parasitic plant invasion [137]. To date, the field application of SL analogs such as GR24, methyl phenlactonoate 3 (MP3), and Nijmegen-1 has shown promising results in combating holoparasitism through a suicidal germination approach [61,131]. Another solution lies in the use of monocot trap crops, as they induce the germination of broomrapes through the release of SLs but, unlike dicotyledons, are not parasitized [138].

Other scientific research proposes the use of gibberellin (GA) agonists as a cheap alternative to natural bioregulators for the control of broomrape seed banks. Thus, GAs have been shown to elicit in broomrape seeds germination activity similar to that of dormant seeds of several autotrophic plant species. The study carried out by Bao et al. (2017) [139] with *P. aegyptiaca* reported that as in non-parasitic angiosperms, seed dormancy was mediated simultaneously by an increase in gibberellic acid and a reduction in abscisic acid (ABA). Finally, GAs applied in vitro to the seeds of *P. ramosa* and *Orobanche* minor promoted germination in the absence of a host or any other germination stimulator [65].

Genetic approaches have demonstrated the importance of ethylene and auxin signaling pathways for proper haustorium formation during sapling invasion. Cui et al. (2020) [140] proposed that parasitic plants use ethylene as a signal to invade host roots. In their research, mutants showing severe haustorial defects were linked to point mutations in homologs of ETHYLENE RESPONSE 1 (ETR1) and ETHYLENE INSENSITIVE 2 (EIN2), which are key signaling components in the response to the gaseous phytohormone ethylene. Additionally, treatment with ethylene signaling inhibitors also caused similar haustorial abnormalities, which indicates that ethylene signaling regulates cell proliferation and differentiation within the parasite. Furthermore, the genetic disruption of ethylene production in the host also disrupted parasite invasion. Numerous studies indicate that auxins play a crucial role in plant–parasite interactions; however, the responses vary among different species and appear to depend on the type of parasitism involved. Ishida et al. (2016) [133] reported that the localized expression of the auxin biosynthesis gene *YUC3* in epidermal cells near the contact site resulted in the accumulation of newly synthesized auxins at the haustorial apex close to the host. This accumulation was necessary and sufficient for priming haustorium formation in the facultative hemiparasitic species *Phtheirospermum japonicum*. By contrast, auxin showed no haustorium induction in germinated seeds of the obligate holoparasite *P. ramosa* and had an inhibitory effect when applied together with root exudates [141]. The translocation of nutrients from the host to the broomrape is facilitated by a continuous vascular system at the host–parasite interface [11]. High levels of auxin are deemed essential for establishing the parasite–host xylem connection, as they interfere with the normal development and differentiation of xylem vessels in *P. ramosa* tubers in infected tomato plants [142]. On the other hand, the disruption of normal auxin transport in strigolactone-deficient mutants resulted in increased susceptibility to infection by parasitic plants [137]. In the terminal haustoria of *Striga asiatica*, the orthologs SOLITARY ROOT (SLR) (INDOL-3-ACETIC ACID INDUCIBLE 14/IAA14) and AUXIN RESPONSE FACTOR 19 (ARF19) were specifically expressed in the early stage of haustorium development [130]. Xiao et al. 2022 reported that the application of an auxin biosynthesis inhibitor increased the rate of haustorium formation and the cessation of cell division in obligate hemiparasite *Striga hermonthica* [143]. Furthermore, the authors of the study proposed that preventing meristem differentiation by applying auxin or a metabolite with a similar effect could be an alternative approach to suicidal germination by preventing prehaustorium formation and, therefore, infestation by the parasite. Finally, Wakatake et al. (2020) demonstrated that the cooperative action of auxin transporters is responsible for controlling xylem vessel connections between the parasite and the host [144]. In their work, they suggest that these connections could be based on the Sachs model, where auxin “sources” (regions of high concentration) connect with “sinks” (regions of low concentration) in a self-organized manner. In parasitic plants, the apex of the haustorium acts as a source, and the site of xylem plate formation near the root vasculature acts as a sink.

The mechanisms leading to haustorium formation remain largely unknown. However, there is evidence that this event is triggered by a cytokinin (CK) signal, which, unlike auxins, is effective in both obligate and facultative parasitic plants. Bioassays by Goyet et al. (2017) demonstrated through the application of exogenous CKs and the specific CK receptor inhibitor PI-55 that CKs exuded by the roots of the host (*Brassica napus*) are crucial for haustorium formation and increase the aggressiveness of *P. ramosa* infection [141]. Roots of broomrape-infested plants were characterized by an enrichment of trans- and cis-Zeatin, which are CKs with high biological activity, and CK nucleosides, which are the predominant transport form in plant vascular tissues [129]. These results highlight that CKs are authentic rhizospheric signals for holoparasites. Furthermore, other studies reported that kinetin, which is a synthetic CK, and 6-benzylaminopurine (BAP) induce prehaustorium-like structures in *Striga asiatica* and *Triphysaria versicolor*, which are obligate and facultative parasites, respectively (reviewed in Goyet et al., 2019 [145]).

Similar to infections by pathogens and herbivores, parasitic plants also induce defense mechanisms in the parasitized host. The most well-studied responses involve systemic acquired resistance (SAR) induced by jasmonic acid (JA) and salicylic acid (SA), which also involve the expression of pathogenesis-related (PR) genes [146]. Abscisic acid (ABA)-mediated responses have also been established [147]. In addition to its prominent role in plant adaptation to abiotic stress conditions, ABA is intrinsically involved in host–holoparasite interactions. For instance, infection with *P. ramosa* resulted in increased levels of ABA and ABA-glucose ester (ABA-GE) in the leaves and roots of wild-type and SL-deficient tomato lines [137]. Another study found that infection of tobacco plants with a mix of *Phelipanche* spp. led to a significant increase in ABA and phaseic acid (PA), a degradation product with biological activity similar to ABA, in root tissues [129]. As part of a common defense-response module, it has been demonstrated that tomato genes related to JA, SA, and ABA are upregulated in tomato plants in the early stages of parasitism by *P. ramose*, and, interestingly, this upregulation is complemented by an increase in the expression of SL biosynthetic genes. Additionally, SL-deficient tomato mutants are described as having reduced levels of JA, SA, and ABA, which points to them as more susceptible to fungal pathogens [148]. So far, research indicates that plants weakened by abiotic factors become more vulnerable to biotic stress factors such as broomrapes; conversely, under abiotic stress conditions, parasitic plants might encounter potential hosts with already-activated SAR. Several reports support the view that hormonal signals induced by abiotic stress may render potential host plants less susceptible or even resistant to parasitism [149,150]. As reported by Foyer et al. (2016) in their research on cross-tolerance, a single stress factor was able to induce different hormonal signaling pathways (SA, JA, ethylene, ABA, and auxins) that led to increased resistance to multiple biotic and abiotic factors [151].

Recently, the results published by Martínez-Melgarejo et al. (2024) align with the information reviewed in the present work [152]. Their research analyzed the comprehensive metabolomic profile of rootstocks that are sensitive and resistant to broomrapes to elucidate the resistance mechanisms conferred by the rootstock and the metabolomic alterations caused by the parasite in the host and vice versa. The results showed that, in samples of sensitive rootstocks, SLs, GAs, and CKs were clustered with the photoassimilates of the plant (sugars, amino acids, and organic acids), which suggests their role in the induction of sink activity in the host root for the development of broomrapes. Conversely, the ethylene precursors ACC, SA, and JA were negatively correlated with factors that favor parasitism, which indicates their participation in the defense against infection and the development of broomrapes.

Ultimately, hormonal signals that enhance tolerance to abiotic stress may also bolster resistance to biotic stress through the upregulation of shared defense mechanisms. Therefore, to accurately assess the effects of host defense mechanisms, it is essential to carefully consider the specific species and environmental conditions of both the host and the parasite.

## 5. Breeding for Resistance

### 5.1. Industrial Tomato

Cultural practices and herbicide application have been commonly used to control *Orobanche* and *Phelipanche* species, though these methods often come with high costs. Breeding for hosts’ resistance offers a more sustainable and cost-effective solution for eliminating this parasitic weed [153]. Cultivated tomatoes are a vital crop and a major nutritious food source for humans, yet many commercial varieties are susceptible to infection by broomrape species. However, knowledge regarding tomato defense mechanisms against broomrape parasitism remains limited. Despite extensive screening efforts, no fully immune strategy or robust resistance to broomrape has been identified [154]. Breeding for broomrape resistance faces challenges, as there is a complex interaction between the host plant and the broomrape species, and there is a need to balance resistance with other favorable agronomic traits in tomatoes [90].

However, several research studies discuss various breeding strategies for managing broomrape resistance in tomato. Most of these breeding strategies follow specific steps: the first step is referred to as a pre-breeding method through the selection and exploitation of genetic material tolerant to Orobanche. According to this approach, it is recommended to explore and exploit sources of natural genetic variability regarding broomrape resistance, which is hidden in tomato wild relatives, traditional varieties, and accessions [155,156]. Indicatively, in a study in 2016, researchers evaluated a collection of 60 tomato accessions consisting of cultivated varieties and wild relatives, screened the response of each genotype to *P. aegyptiaca* infection, and highlighted that screening and selection procedures can be considered critical steps in pre-breeding to prioritize genetic material for further breeding purposes [157]. The second step is conventional/classical breeding strategies, which can be based on the pre-breeding method mentioned above. Traditional breeding techniques, such as hybridization and backcrossing with traditional or wild tomato genetic materials, can effectively introgress favorable traits to *S. lycopersicum*. While these methods are time-consuming, they enable the transfer of robust genetic resistance from unexplored genetic material to cultivated tomato varieties [157,158]. In 2020, Ghani et al. developed intraspecific hybrids using *S. lycopersicum* and two wild relatives, *S. pimpinellifolium* and *Solanum habrochaites*, which are known for their resistance against various abiotic and biotic stresses and other agronomic traits, noting their potential use for broomrape tolerance in tomato [159]. Investigating host resistance mechanisms is also proposed as an important approach for evolving breeding strategies in tomatoes. Regarding resistance against *P. aegyptiaca*, *O. cernua*, and *P. ramosa*, a plethora of tomato varieties has been investigated; however, only a moderate level of resistance has been observed [90,160,161,162]. Rubiales examined the different resistance mechanisms in host tomato plants, including pre-attachment and post-attachment resistance, and how these can vary across broomrape species [163]. To this end, Marker-Assisted Selection (MAS) would be highly preferable as a potential method for tomato breeding against broomrape parasitism to efficiently select plants with broomrape-resistant characteristics, speeding up the breeding process [153,157,164]; however, in the existing literature, the available data about specific applications are still very few, mainly focusing on functional markers [148].

Several research works also highlight the importance of Quantitative Trait Loci (QTL) mapping to identify genetic regions associated with broomrape resistance [164]. Bai et al. (2020) investigated resistance to *P. aegyptiaca* in tomatoes by assessing 76 introgression lines (ILs) derived from *S. pennellii* LA0716. They identified 13 QTLs associated with various resistance traits distributed across several chromosomes. Specifically, IL6-2 and IL6-3 possessed QTLs located on chromosome 6, demonstrating profound tolerance to the holoparasite [165]. These findings are in accordance with recently published results in 2024 on early-stage parasitism by *P. ramosa* in these two ILs, which, in comparison to a susceptible commercial *S. lycopersicum* hybrid, revealed remarkable differences in the expression of genes found in several chromosomic regions, including chromosome 6, which is highly related to biotic stressors [154].

Conventional mutagenesis and TILLING (Targeting Induced Local Lesions In Genomes) are valuable alternatives for breeding against broomrape parasitism. Strigolactones (SLs) are plant hormones that play a crucial role in various plant processes, including the stimulation of seed germination in parasitic plants like *Phelipanche* and *Orobanche* species. Koltai et al. (2010) produced through fast-neutron mutagenesis a tomato line resistant to broomrape, named Sl-ORT1 (*S. lycopersicum* Orobanche Resistant Trait 1). In that specific line, they noticed that low transcript levels of the *SlCCD7* gene were involved in strigolactones’ (SLs) biosynthesis [166]. TILLING is a technique that can be used for the identification and characterization of specific mutations within genes. In 2018, Hasegawa et al. used two tomato TILLING genotypes (from the Micro-Tom tomato variety) deficient in the *CAROTENOID CLEAVAGE DIOXYGENASE 8* (*CCD8*) gene (which encodes enzymes that play a crucial role in the biosynthesis of SLs), and they studied its relevant expression levels, revealing that the mutants downregulated the expression of the *CCD8* gene, highlighting the potential role of *CCD8* in broomrape-resistant mechanisms [167].

Stacking multiple resistance genes and engineering biochemical pathways by implementing new breeding approaches and biotechnological tools is an emerging strategy [153,164]. Research utilizing molecular and biotechnological techniques has exploited the key role of strigolactones (SLs), suggesting that manipulating strigolactone pathways could be an effective strategy for developing crop varieties resistant to parasitic weeds [168,169,170]. RNA-interference (RNAi) is a method widely used in tomatoes to explore SLs’ resistance mechanisms. More specifically, silencing of the *CCD7* and *CCD8* genes, as mentioned above, has been applied, leading to the phenotype’s severe alteration [171,172]. Additionally, the expression of a multi-sequence RNAi construct designed to target three critical *P. aegyptiaca* genes (*PaACS*, *PaM6PR*, and *PaPrx1*) demonstrated resistance to broomrape infestation [173]. Moreover, other molecular studies utilizing omic approaches indicate that, in addition to the essential role of SL-induced resistance mechanisms, multiple cellular processes may be involved in the tomato’s response to broomrapes [154].

Genome editing and transgenic approaches have also been proposed for broomrape management. Research highlights that CRISPR/Cas and other gene-editing technologies are creating new opportunities for targeted modifications in vegetable genomes, potentially strengthening resistance traits that traditional breeding cannot easily achieve. Several studies have applied transgenic approaches to introduce resistance genes from other species. More specifically, in 2006, Radi et al studied the expression of the *sarcotoxin IA* gene—which is a gene originally identified in insects, characterized by antimicrobial activity and the fact that when it is expressed in plants, it provokes resistance against pathogens—driven by a root-specific promoter (tob promoter) in tomato plants. The genetic modification enhanced the resistance of tomato plants against broomrape species [174]. Genome editing techniques, like knocking out genes relevant to SL biosynthesis (like *CCD8*, *MAX1*, *P450*, and *CYP722C*) from tomato genotypes, resulted in resistance against several broomrape species and different levels of pleiotropy on the phenotype [175,176,177,178]. While these methods are very promising, regulatory challenges and consumer acceptance continue to limit the broad use of transgenic/edited plants for broomrape resistance [153,164].

**Figure 2:**
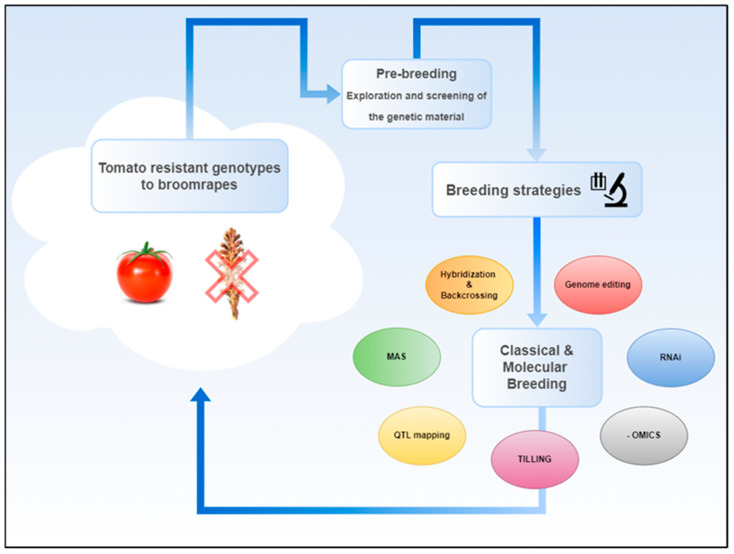
A holistic approach to breeding strategies to create tomato resistant genotypes to broomrapes.

Overall, various studies recommend a combination of the aforementioned breeding strategies, emphasizing the importance of integrating both classical and modern breeding techniques to develop durable resistance in tomato varieties and achieve long-term solutions to broomrape infestations in tomato crops [155,158,163] (Figure 2).

### 5.2. Legumes

Investigations regarding broomrape resistance have included major legumes, like faba bean, chickpeas, peas, and lentils, although most research outcomes have arisen from research on faba bean.

Faba bean (*Vicia faba* L.) is one of the most important legume crops worldwide, ranking fourth with respect to total production after chickpea (*Cicer arietinum* L.), field pea (*Pisum sativum* L.), and lentil (*Lens culinaris* L.) [179,180]. It is cultivated extensively on all continents, with the largest producers being China, Ethiopia, the United Kingdom, and Australia, and it constitutes a major grain legume in the Mediterranean basin that is used in the human diet and as animal feed, given its high protein content (FAOSTAT, 2023). In Tunisia, it represents a predominant crop accounting for about 75% of total grain legumes, with about 60 k ha and about 400 k tons [181,182], and it is of equal importance in Egypt, Algeria, and Morocco (FAO 2021) [183]. However, its productivity in the Mediterranean area has been unstable, with significant fluctuations in agricultural yield due to a range of factors, including abiotic and biotic stresses related to the severe climate change in the region. One of the main challenging abiotic factors is the elevated temperature and drought in the area over the last several years, as well as the catastrophic effect of infestations by holoparasitic weeds of the Orobancaceae family. Especially in Tunisia, broomrape infestations are mainly caused by four species: *O. crenata*, *O. foetida*, *O. cumana*/*cernua*, and *P. ramosa*/*aegyptiaca*. Among these, *O. foetida* poses the most severe threat; with yield losses in faba beans reaching up to 90% [18,184,185], the difficulty of managing this parasite resides in its exceptional fertility, which gives rise to about 50,000 to 500,000 seeds/plant [18].

A recent report on a population structure and genetic diversity analysis based on dominant marker ‘RAPD’ revealed a significant genetic disparity within individuals of each of the three studied genera and species *O. foetida*, *O. crenata*, and *P. ramosa* [185]. In this context, the three species were clustered into two main metapopulations and then into two genetic groups based on genus and species diversity levels. There was no geographic origin correlation as a consequence of the low levels of diversity between the populations; the breeding schemes for the introgression of resistance to grain legumes against broomrapes can be conducted in one location [185]. In other reports, it was shown that *O. foetida* Poir. has two different varieties; one in North Africa, var. foetida in Tunisia, and var. Broteri in Morocco and Spain [186]. Regarding developing legume varieties resistant to broomrapes, previous studies have shown that the resistance can be broken by the mutation of the parasite itself, which is a significant challenge for breeders [25].

Numerous strategies have been adopted over the years aimed at managing Orobanche parasitism, including mechanical, chemical, and biological control as well as cultivation practices. However, the capacity of these methods is limited in providing full protection to the host plant against Orobanche attacks. Another effective approach that holds promise and could work in synergy with the above constraining methods is the development of broomrape-resistant varieties through breeding programs.

Legume cultivars showing low susceptibility/partial resistance to Orobanche infestations constitute a valuable genetic resource for studying and understanding defense mechanisms and designing crop improvement programs toward superior genotypes with increased resistance to the parasite. Along these lines, the characterization of different faba bean varieties, breeding lines, and mutant lines with partial resistance to *Orobanche* and *Phelipanche* species highlights research efforts focusing on developing genotypes that harbor efficient resistance mechanisms to counteract broomrape attacks [5,164]. Distinct faba bean varieties derived mostly from the Egyptian donor Giza402 showed different degrees of resistance to *O. crenata*, which was most likely linked to the prevention of parasite penetration through the vasculature structure of the host [187]. Experiments with two breeding lines, ‘Quijote’ and ‘Navio’, recorded low levels of *O. crenate*, *O. foetida*, and *P. aegyptica* seed germination, possibly owing to the decreased secretion of germination stimulants in root exudates [187,188]. The study of six faba bean accessions with partial resistance against various broomrape species indicated diversified resistance involving pre- and post-attachment resistance-related processes [189]. Similarly, an evaluation of a series of genotypes in field and controlled conditions identified Giza 843, Misr1, and Misr3 as genotypes with good resistance to *O. crenata* and with significantly higher yields than the susceptible ones. Based on the low effects of parasitism on the yield and vegetative growth of host plants, Giza 843 was selected as a promising candidate genotype to be introduced into Moroccan faba bean breeding programs [190]. Recently, the Tunisian national faba bean breeding program, led by the field crop laboratory of INRAT in collaboration with the CRRGC (the Regional Field Crops Research Center of Beja), registered new partially resistant varieties to *O. foetida* and *O. crenata* in faba beans. These new cultivars belong to the small, seeded type, named “Chourouk” (i.e., with regular tannin) and “Zaher” (i.e., with low tannin), and the medium-seeded type, named “Chams”, and have good field performance and broomrape resistance compared to the susceptible varieties examined [18,78,79]. In this context, the first faba bean candidate variety (named Ammar) was deposited for registration in the Tunisian official national catalogue; field trials are to be performed in 2024 and 2025 in a multi-site field trial (Khamassi 2024, personal communication). All the above-mentioned varieties showed a number of distinguished characteristics, such as low *Orobanche* spp. seed germination stimulant production, low numbers of parasite seed germination, restricted numbers of haustorium attachment, and decreased growth of established tubercles; hence, they resulted in a low number of final emerged shoots of Orobanche in the host plant [191,192,193,194,195].

Moreover, faba bean mutant lines were generated by gamma irradiation of ‘Badi’, which is highly susceptible variety to *O. crenata* and *O. foetida*. Lower levels of parasite seed germination were detected for these lines, which suggests that developing resistant genotypes through mutagenesis may be plausible. However, this genetic material is still under study, facing registration challenges [196]. Similar studies have been undertaken to explore the potential of pea germplasm in developing effective resistance to Orobanche infestations. A pea landrace (ROR12) known to guarantee good yields even in heavily infected fields was found to exhibit high levels of resistance to *O. crenata*, most likely due to interference with the parasite development process leading to the delayed emergence of the weed. In addition, ROR12 root exudates displayed decreased strigolactone (SL) accumulation, and their ability to stimulate germination of *O. crenata* seeds in vitro was significantly reduced. Interestingly, induction of the *RAMOSUS1* (*RMS1*) gene encoding the CCD7 enzyme involved in SL biosynthesis was decreased in the infected host plants, which points to the altered regulation of the SL biosynthetic pathway upon parasitism that may be associated with the establishment of resistance to *O. crenata* [197]. Considering the above observations, the ROR12 landrace could serve as a promising candidate for cultivation as well as introduction in breeding programs aiming to develop Orobanche-resistant pea genotypes. Breeding efforts involving various crosses among *P. fulvum*, *P. sativum* ssp. *elatius*, *P. sativum* ssp. *Syriacum*, and pea landraces led to the development of pea lines with partial resistance to *O. crenata*. Resistance was associated with reduced tubercle formation both in rhizotron and field conditions, and the derived pea lines displayed good performance and relatively high yields in *O. crenata*-infected fields [5].

Delineating host–parasite interactions at the genetic–molecular level has lagged behind in the case of commercial legume crops as compared to tomato, where ample omics and gene editing approaches have become feasible nowadays. However, recent transcriptomic findings in faba bean have begun to illuminate the molecular mechanisms underlying legume responses to parasitic infection and the molecular basis of resistance to Orobanche. An elaborate RNA-seq approach was deployed using a susceptible faba bean variety (Badii) and a resistant variety (Chourouk) at three developmental stages of *O. foetida* development (before germination, after germination, and tubercle stage), aiming to unravel the intricate molecular processes underlying susceptibility/resistance in the faba bean/*O. foetida* interaction [198]. The transcriptomic profile revealed differential gene expression between the resistant and susceptible genotypes and among the different parasitism stages in pathways associated with secondary metabolites such as flavonoids, auxin, thiamine, and jasmonic acid. Importantly, differences in gene expression were observed in genes involved in the orobanchol biosynthesis pathway, such as the *MAX1*, *MAX1B*, and *CYP711A3* homologs and, in particular, the key gene *CYP722C*. Notably, knocking out this gene in tomato using CRISPR/Cas9–mediated genome editing resulted in undetectable orobanchol in tomato roots, whereas the architecture of the edited plant remained unaltered [178], which suggests that *CYP722C* could serve as a good target for developing resistant varieties to broomrapes.

## 6. Conclusions

### 6.1. Management Strategies

The management of broomrapes in Mediterranean cropping systems requires a strategic combination of various preventive and curative measures with the goal of reducing the infestation and minimizing the impact of the parasite on crop yield and quality. Preventive methods are the cornerstone of broomrape management because if the parasite is established, it becomes difficult to control. Preventive measures focus on disrupting the parasite’s life cycle and reducing the chances of infestation. There is a plethora of data for a long list of preventive methods such as rotations including trap, catch, or allelopathic crops; intercropping; utilizing winter cover crops; modifying sowing or transplanting dates; solarization; tillage or mechanical cultivation; fertilization; the use of synthetic stimulants for suicidal germination; polyethylene plastic mulching; and the use of crop tolerance inducers. Curative methods for broomrapes play a smaller role, and the focus is on reducing their impact on the current crop. These measures generally aim to manage or limit the spread and damage caused by the parasite using the following tactics: seed dressing treatments, foliar herbicides, herbigation, biological control agents, and hand weeding. The integrated management of broomrapes involves combining preventive and curative measures in a way that maximizes their effectiveness. A good example of a combined strategy is using a partially resistant faba bean variety (i.e., Hashenge), using bio-inoculants (*R. leguminosarum* and *Trichoderma harzianum*), and performing hand weeding at the crop flowering stage [85]. This integrated management strategy, when implemented together, has been shown to enhance the productivity of the crop while also reducing the impact of the parasitic weed.

### 6.2. Recognition of Parasitic Plant Molecular Patterns by Hosts

The recognition of parasitic plant molecular patterns by hosts underscores the complexity and significance of host–parasite interactions in plant ecology and evolution. Understanding these interactions on a molecular level is crucial, as parasitic plants can significantly impact agricultural productivity and ecosystem dynamics. Host plants have evolved sophisticated mechanisms to recognize and respond to parasitic threats [123]. Primarily through the detection of pathogen-associated molecular patterns (PAMPs) unique to the parasites, host plants are able to recognize alien invaders.

The recognition of these patterns by host plants triggers a series of defense responses [104] that can halt or mitigate the parasitic invasion. This complex immune response is akin to the pattern recognition mechanisms observed in plant–pathogen interactions and highlights the co-evolutionary arms race between hosts and their parasitic adversaries.

Advancements in molecular biology and genomic technologies have significantly enhanced our capacity to identify these molecular patterns and understand the underlying recognition mechanisms. Unraveling these interactions contributes to our broader understanding of plant immunity and the evolutionary dynamics between species. Additionally, such insights could lead to the development of crops with improved resistance to parasitic plants, thereby enhancing food security and sustainable agricultural practices. Therefore, continued exploration of these interactions will not only advance our knowledge of plant biology but also offer new strategies for managing parasitic plant impacts in natural and agricultural ecosystems.

### 6.3. Hormonal Crosstalk Between Crop and Parasite

Plant hormones play a fundamental role in interactions between parasitic and crop plants, regulating processes from seed germination in parasitic plants to haustorium formation and host invasion. Strigolactones (SLs) are key chemical signals that are essential for the germination of parasitic seeds because they indicate the proximity of the host, while auxins, gibberellins, and ethylene are fundamental for the vascular connection between the parasite and the host, facilitating the flow of nutrients. Additionally, cytokinins (CKs) exuded by the host roots are crucial for haustorium formation, increasing the aggressiveness of the infection. Other defense hormones such as jasmonic acid (JA), salicylic acid (SA), and abscisic acid (ABA) play a key role in the host response to parasitism, activating resistance mechanisms to prevent infections. Understanding how plant hormones mediate parasitic relationships will facilitate the development of more effective control strategies, such as the use of SL analogues or monocot trap crops. However, many of the hormonal mechanisms involved remain unclear, which poses a challenge for optimizing parasitic plant management.

### 6.4. Breeding for Resistance

Research on broomrape resistance in tomato and legumes emphasizes the development of sustainable and cost-effective solutions by combining breeding strategies and techniques. In tomato species, the breeding target is to utilize both conventional methods and advanced molecular techniques to introduce favorable resistant traits. The challenge of these procedures is to keep the balance between resistance to broomrapes and other agronomic traits due to the complex host–parasite interactions. Similarly, in legumes like faba beans, breeding programs have identified the developed resistant varieties through genetic diversity analysis and traditional breeding approaches [163]. To conclude all the above, the exploitation of plant breeding strategies and the combination of classical and modern techniques are crucial for achieving durable resistance and ensuring long-term crop productivity and sustainability against broomrape infestations.

## Figures and Tables

**Table 1:** Current status of the list of preventive and curative techniques for controlling broomrapes in major Mediterranean crops such as legumes and industrial tomato. The efficacy levels are based on expert estimation after reviewing the related literature. The mechanism and the primary effects of the techniques refer to parasite life cycle and broomrape stage targeted.

Technique	Efficacy Levels ^(a)^		Mechanism; Primary Effects
	Legumes	Ind. Tomato	
I. Preventive			
Rotation	+	+	(M): Disruption of the parasite life cycle by avoiding soil seed bank replenishing (E): Soil seed bank; seed germination; pre- and early attachment of haustorium
Winter cover crops	na ^1^	+	(M): Disruption of the parasite life cycle by creating a hostile soil environment (E): Soil seed bank; seed germination; pre-attachment of haustorium
Use of trap, catch, or allelopathic crops; intercropping	+	+	(M): Disruption of the parasite life cycle by reducing final haustorium numbers (multiple tactics) (E): Soil seed bank; seed germination; pre-attachment of haustorium
Modify sowing, transplanting dates	+	+	(M): Disruption of best conditions for the onset of parasitism (E): Soil seed bank; seed germination; pre- and early attachment of haustorium
Solarization	+++	na ^2^	(M): Reduction of active soil seed bank by seed decay (E): Soil seed bank; seed germination
Tillage; mechanical cultivation	++	++	(M): Reduction of active soil seed bank by physical misplacement of seeds (E): Soil seed bank; seed germination
Fertilization	+	++	(M): Disruption of the parasite life cycle by employing direct (on the parasite seeds) and indirect (enhancing crop competitive ability) conditions (E): Soil seed bank; seed germination; pre-, early, and late attachment of haustorium
Use of synthetic stimulants	+	+	(M): Lower the onset of parasitism using suicidal germination tactics (E): Soil seed bank; seed germination
Polyethylene plastic mulching	na ^3^	+++	(M): Disruption of the parasite life cycle using physical barriers (E): Seed germination; pre-, early, and late attachment of haustorium
Use of crop tolerance inducers	+	+	(M): Disruption of the parasite life cycle by enhancing crop competitive ability (E): Seed germination; pre- and early attachment of haustorium
Use of resistant/tolerant cvs	++	na ^4^	(M): Lower or minimize onset of parasitism by natural crop tolerance/resistance development (E): Seed germination; pre- and early attachment of haustorium
II. Curative			
Seed dressing treatments	++	++	(M): Lower onset of parasitism by utilizing synthetic substances applied as seed coating, film, or pellets (E): Seed germination; pre- and early attachment of haustorium
Foliar herbicides	++	+	(M): Lower onset of parasitism by utilizing herbicides to reach target sites via translocation of the haustorium (E): Seed germination; pre- and early attachment of haustorium
Herbigation	na ^5^	++	(M): Lower onset of parasitism by utilizing herbicides to be applied with irrigation (E): Seed germination; pre-, early, and late attachment of haustorium
Biological control; beneficial insects	na ^6^		(M): Disruption of the parasite life cycle by biological agents (multiple tactics) (E): Soil seed bank; seed germination; pre- and early attachment of haustorium
Hand weeding	+++	+++	(M): Disruption of the parasite life cycle by physical weed removal (E): Soil seed bank; late attachment of haustorium

^(a)^: Efficacy levels: + low level; ++ medium level; +++ high level. ^1^: Not applicable—No timing window for employing winter cover crops. ^2^: Not applicable—No timing window for employing solarization in summer crops unless fallow. ^3^: Not applicable—Such a cropping system does not exist for legumes. ^4^: Not applicable—No current commercial resistant or tolerant cvs available for industrial tomato (only research outcomes). ^5^: Not applicable—Such a strategy (application of herbicides using drip irrigation) does not exist for legumes. ^6^: Not applicable—No current commercial biological control agent available for industrial tomato (only research outcomes).
